# Brain-Derived Neurotrophic Factor Mediated Perfluorooctane Sulfonate Induced-Neurotoxicity via Epigenetics Regulation in SK-N-SH Cells

**DOI:** 10.3390/ijms18040893

**Published:** 2017-04-24

**Authors:** Xin-Xin Guo, Qing-Zhi He, Wu Li, Ding-Xin Long, Xiao-Yuan Pan, Cong Chen, Huai-Cai Zeng

**Affiliations:** 1 Department of Preventive Medicine, School of Public Health, University of South China, Hengyang 421001, China; guoxinxin1677@gmail.com (X.-X.G.); liwu199103@gmail.com (W.L.); longdx@usc.edu.cn (D.-X.L.); panxiaoyuan199104@gmail.com (X.-Y.P.); chencong199206@gmail.com (C.C.); 2 School of Pharmacy and Biology, University of South China, Hengyang 421001, China; heqingzhi666@gmail.com; 3 Hunan Province Cooperative Innovation Center for Molecular Target New Drug Study, Hengyang 421001, China

**Keywords:** PFOS, BDNF, DNMTs, microRNA, DNA methylation

## Abstract

Perfluorooctane sulfonate (PFOS), a new kind of persistent organic pollutant, is widely distributed in the environment and exists in various organisms, where it is also a neurotoxic compound. However, the potential mechanism of its neurotoxicity is still unclear. To examine the role of epigenetics in the neurotoxicity induced by PFOS, SK-N-SH cells were treated with different concentrations of PFOS or control medium (0.1% DMSO) for 48 h. The mRNA levels of DNA methyltransferases (DNMTs) and Brain-derived neurotrophic factor (BDNF), microRNA-16, microRNA-22, and microRNA-30a-5p were detected by Quantitative PCR (QPCR). Enzyme Linked Immunosorbent Assay (ELISA) was used to measure the protein levels of BDNF, and a western blot was applied to analyze the protein levels of DNMTs. Bisulfite sequencing PCR (BSP) was used to detect the methylation status of the BDNF promoter I and IV. Results of MTT assays indicated that treatment with PFOS could lead to a significant decrease of cell viability, and the treated cells became shrunk. In addition, PFOS exposure decreased the expression of BDNF at mRNA and protein levels, increased the expression of microRNA-16, microRNA-22, microRNA-30a-5p, and decreased the expression of DNMT1 at mRNA and protein levels, but increased the expression of DNMT3b at mRNA and protein levels. Our results also demonstrate that PFOS exposure changes the methylation status of BDNF promoter I and IV. The findings of the present study suggest that methylation regulation of *BDNF* gene promoter and increases of BDNF-related-microRNA might underlie the mechanisms of PFOS-induced neurotoxicity.

## 1. Introduction

Perfluorooctane sulfonate (PFOS) is one class of the representative compounds and the ultimate metabolites of perfluorinated compounds (PFCs). Due to its exceptional hydrophobic and oleophobic properties, PFOS was widely used in industrial, commercial, and household applications. PFOS is found in carpets, textiles, leather, paper, fire-fighting foams, and food packing materials, and it is a component of pharmaceuticals and insecticides [1]. However, due to their chemical and thermal stability, PFOS can exist in the environment for a long time, and is highly resistant to microbial degradation [2]. Recent reports showed that concentrations of PFOS in fish reached the level of 15,000 ng/g [3]. PFOS, which is considered to be a new kind of persistent organic pollutant, has the potential adverse effect on the reproductive system, liver, heart, and lung [4]. Especially it also could cause injury to the ability of memory and locomotor function and induce the neurotoxicity [5]. Unfortunately, the underlying mechanism of its neurotoxicity is still unclear. Epigenetic modulation, which can modify the gene expression without DNA sequence alteration, has many layers of complexity, including DNA methylation, histone modifications, chromatin remodeling, and change in expression of microRNAs, as well as long no-coding RNAs [6]. It was reported that the epigenetic regulation played an important role in neurodevelopment and neurobehavioral functions, including learning and memory [7].

Brain-derived neurotrophic factor (BDNF), a key member of the neurotrophin family, plays an important role in neuronal proliferation, synaptic function, and synaptic plasticity through combination with the tropomyosin-related kinase B (TrkB) receptor. After combining with TrkB, BDNF can activate intracellular signaling cascades and affect neuronal biological processes, including cell survival, synaptic structure, and synaptic plasticity [8,9]. Previous studies revealed that rat prenatal PFOS exposure decreased protein expression of BDNF on postnatal day (PND) 35 in the hippocampus tissue of the offspring, but increased the mRNA levels of BDNF [10]. Our previous studies also suggested that PFOS could reduce mRNA and protein levels of BDNF in SH-SY5Y cells [11]. However, the exact mechanism is not very clear and needs more investigation.

MicroRNAs (miRNAs) are evolutionarily conserved, small, and noncoding RNAs. Through binding to the imperfect sequence of target mRNAs, miRNAs result in translational inhibition or destabilization of the target mRNA [12]. The effect of a particular miRNAs on the posttranscriptional control of BDNF expression is beginning to be elucidated [13]. Several studies reported that miRNAs could directly regulate the expression of BDNF [13,14]. For example, micro-30a-5p might target the 3′UTR of BDNF and down-regulate protein expression of BDNF in humans [15]. And microRNA-22 negatively regulated the expression of CREB binding protein [16]. Muiños-Gimeno M reported that microRNA-16 and microRNA-22 can inhibit the expression of BDNF [17]. Therefore, we hypothesize that these microRNAs are involved in PFOS-induced neurotoxicity caused by dysregulation of BDNF.

DNA methylation, one way of epigenetic modulation, is a process by which methyl groups are added to the cytosine base in the dinucleotide sequence 5′CpG3′. Methylation can change the expression of a certain gene without changing its sequence [18]. DNMTs, including DNMT1, DNMT2, and DNMT3, play an important role in the progress of DNA methylation. DNMT3 is divided into DNMT3a and DNMT3b, which participate in the de novo methylation and are involved during early development [19]. Guerrero-Preston R reported that global DNA methylation was associated with in utero exposure to cotinine and perfluorinated alkyl compounds [20]. PFOS could decrease global DNA methylation and methylation of the LINE-1 regulatory region, but increase the GSTP promoter region methylation [21]. These studies show that PFOS can change the DNA methylation level. Therefore, we assume that PFOS could lead to the CpG methylation of BDNF mediated by DNMTs and decrease the expression of BDNF.

The human neuroblastoma cell line, SK-N-SH, is widely used to determine the neurotoxicity of xenobiotics, as well as to characterize essential features of the neurotoxicity of different compounds such as metals, enterovirus A71, and arsenic trioxide [22,23]. Based on the evidence, to better understand the underlying molecular mechanisms of neurotoxicity of PFOS, the present study evaluated the general cytotoxicity of PFOS and investigated the effects of PFOS on the BDNF expression in SK-N-SH cells. Additionally, this study explored the mechanism that PFOS affected BDNF expression via miRNA and methylation regulation.

## 2. Results

### 2.1. Morphology Analysis of SK-N-SH Cell after PFOS Exposure

**Figure 1:**
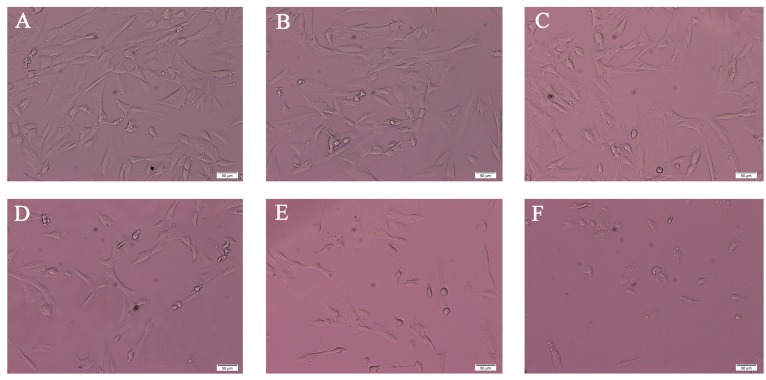
Effects of PFOS on SK-N-SH cell morphology. SK-N-SH cell morphology was observed after a 48-h exposure to various concentrations of PFOS. (**A**) Control; (**B**) 50 μM PFOS; (**C**) 100 μM PFOS; (**D**) 150 μM PFOS; (**E**) 200 μM PFOS; (**F**) 250 μM PFOS. (Bar: 50 μm).

To identify the effect of PFOS on SK-N-SH cell morphology, cells were treated with different concentrations of PFOS or DMSO (control) for 48 h. As shown in Figure 1, with the increase of concentration of PFOS, compared with control, neurites of SK-N-SH became less and less, and cells shrunk and became round.

### 2.2. PFOS Inhibited Cell Growth in SK-N-SH Cells

**Figure 2:**
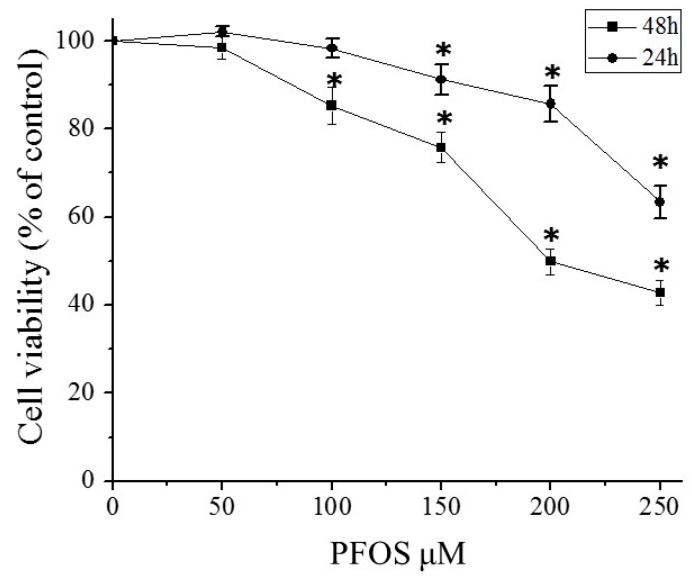
Effects of PFOS on SK-N-SH cell viability. Cell viability was determined by MTT assay after 24 or 48-h exposure to various concentrations of PFOS (50, 100, 150, 200, 250 μM) or DMSO (control). Data are presented as mean ± SD of three separate experiments. * Compared with control, respectively: *p* < 0.05.

As shown in Figure 2, the MTT assay showed that when SK-N-SH cells were exposed to PFOS at 150 μM for 48 h, the percentage of viable cells was reduced to 75% compared with the control group. With the increase of the concentration of PFOS, the cell activities gradually declined. When the PFOS concentration reached 250 μM, the viability of cells reduced to 42.8% (*p* < 0.05).

### 2.3. PFOS Reduced the Expression of BDNF

**Figure 3:**
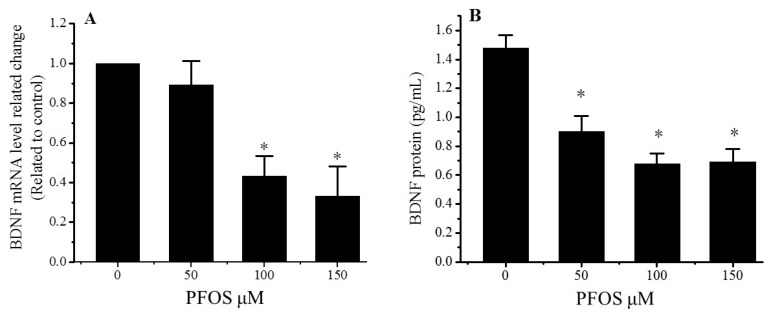
Effects of PFOS on the expression of brain-derived neurotrophic factor (BDNF), in SK-N-SH cells. (**A**) BDNF mRNA levels in SH-SY5Y cells. Each data point was normalized to the control (DMSO). (**B**) BDNF protein levels in SH-SY5Y cells. The data are presented as mean ± SD from three independent experiments. * Compared with control: *p* < 0.05.

The mRNA and protein levels of the BDNF in the SK-N-SH cells were measured after a 48-h exposure to different concentrations of PFOS. The mRNA level was tested by a QPCR and protein levels were detected by ELISA. The results show that the expression of BDNF mRNA and proteins significantly decreased in the PFOS exposed group (*p* < 0.05, Figure 3). Compared with the controls, the BDNF mRNA levels decreased to 43.3% and 32.2% in the 100 and 150 μM PFOS treatment groups, respectively, after a 48-h incubation (Figure 3A). There is a significant difference between the control group and the PFOS exposed groups. When the concentration of PFOS reached 150 μM, the levels of BDNF protein declined to 0.69 pg/mL (Figure 3B).

### 2.4. The Effects of PFOS on the BDNF Promoter Methylation in SK-N-SH Cells

**Figure 4:**
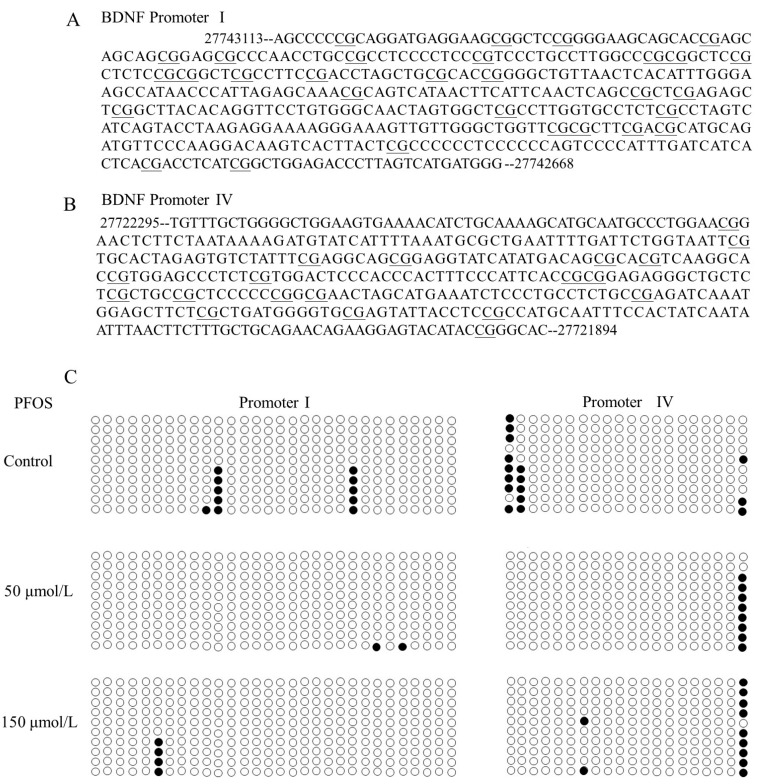
Bisulfite-sequencing methylation analysis of individual CpG dinucleotides of the BDNF promoter I (**A**) and IV (**B**) from the SK-N-SH Cells. (**A**) Locations of these 30 CpG sites within promoter I are listed and the methylation status was assessed by bisulfite sequencing. (**B**) Locations of these 20 CpG sites within promoter IV are listed and the methylation status was assessed by bisulfite sequencing. (**C**) Methylation of promoter. White and black circles denote unmethylated and methylated CpG sites, respectively. Each row indicates a specific plasmid clone. Ten clones came from the same group. The underline in the image (**A**,**B**) represent CpG dinucleotides site.

DNA methylation is a known regulator of gene expression. We examined methylation of the *BDNF* gene promoter I and IV. The DNA methylation was confirmed using a bisulfite-sequencing PCR, which examined the methylation of 30 CpG dinucleotides of promoter I and 20 CpG dinucleotides of promoter IV. The results show that the methylated CpG sites of the BDNF promoter I and IV (Figure 4). In promoter I, the averages of methylation frequency of 10 clones in three groups were 3.7%, 0.7%, and 1.3%, respectively. In promoter IV, the averages of methylation frequency of 10 clones in three groups were 8.0%, 4.0%, and 5.0%, respectively. Interestingly, PFOS lead the high methylation status of the twentieth CpG site with promoter IV. Methylation frequencies of this CpG site were 30%, 80%, and 90% in control group, 50 μmol/L group, and 150 μmol/L group, respectively.

### 2.5. Effect of PFOS on the Expression of DNMT1, DNMT3a, and DNMT3b in SK-N-SH Cells

**Figure 5:**
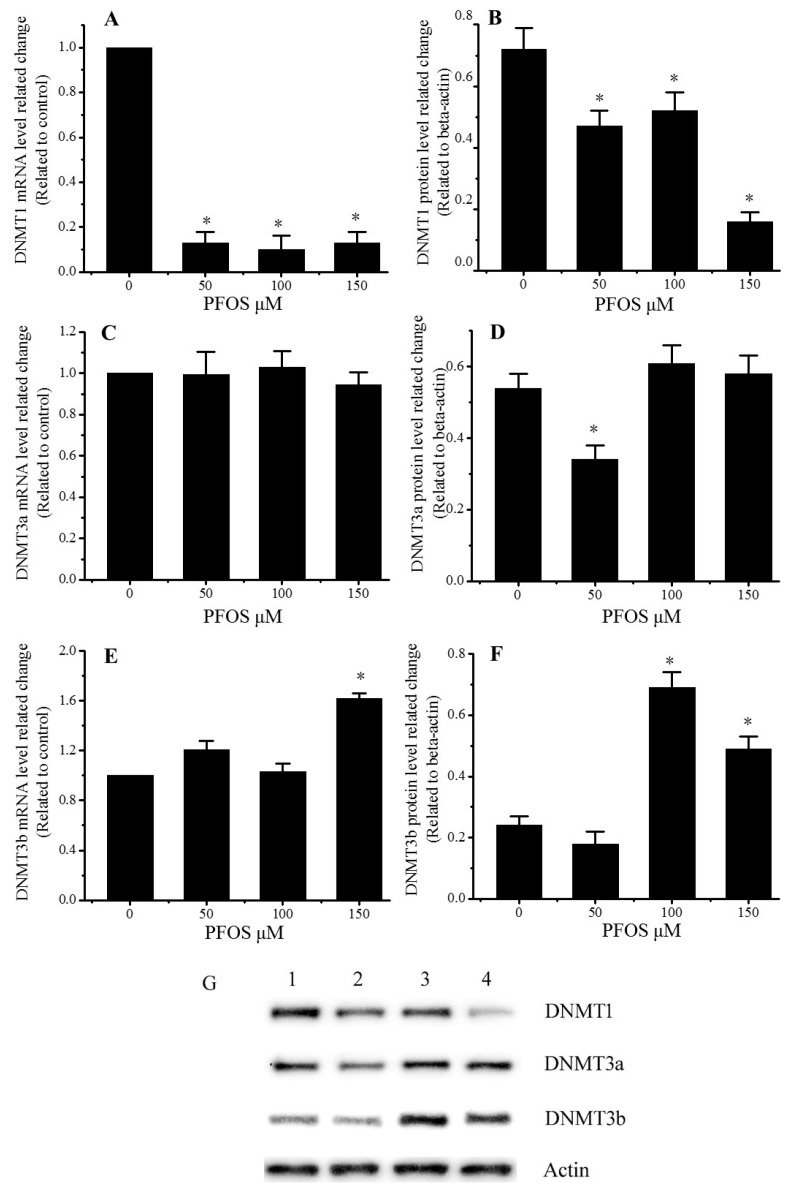
Effects of PFOS on the expression of DNMTs in SK-N-SH cells. Cells were cultured for 48 h with various concentrations of PFOS (50, 100, 150, 200 μM) or DMSO (control). (**A**) DNMT1 mRNA levels in SK-N-SH cells; (**B**) DNMT1 protein levels in SK-N-SH cells; (**C**) DNMT3a mRNA levels in SK-N-SH cells; (**D**) DNMT3a protein levels in SK-N-SH cells; (**E**) DNMT3b mRNA levels in SK-N-SH cells; (**F**) DNMT3b protein levels in SK-N-SH cells; (**G**) Protein lane of DNMT1, DNMT3a, DNMT3b, and β-actin. Lane 1, control; lane 2, 50 μM PFOS; lane 3, 100 μM PFOS; lane 4, 150 μM PFOS. The data are presented as mean ± SD from three independent experiments. * Compared with control: *p* < 0.05.

As shown in Figure 5, PFOS could decrease the expression of DNMT1, and increase the expression of DNMT3b. There are no significant differences between the control group and the PFOS groups about the mRNA level of DNMT3a, but compared with control group, the protein level of DNMT3a decreased for the 50 μM PFOS group.

### 2.6. PFOS Increases the Expression of MicroRNA-16, MicroRNA-22, and MicroRNA-30a-5p

**Figure 6:**
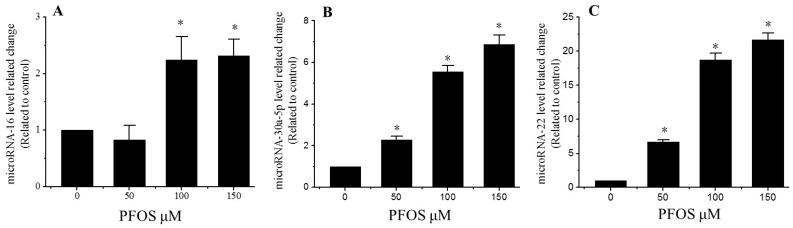
Effects of PFOS on the expression of mRNAs in SK-N-SH cells. Cells were cultured for 48 h with various concentrations of PFOS (50, 100, 150, 200 μM) or DMSO (control). (**A**) mRNA levels of microRNA-16 in SK-N-SH cells; (**B**) mRNA levels of microRNA-30a-5p in SK-N-SH cells; (**C**) mRNA levels of microRNA-22 in SK-N-SH cells. Each data point was normalized to the control (DMSO), and the data are presented as mean ± SD from three independent experiments. * Compared with control: *p* < 0.05.

Many previous studies have shown that microRNAs can regulate the expression of BDNF [11]. To better identify the mechanism how PFOS inhibited the expression of BDNF through microRNAs. The relative expression of BDNF-related microRNA-16, microRNA-22, microRNA-30a-5p, were measured by QPCR. As shown in Figure 6, compared with the control, the relative expression of microRNA-16, microRNA-22, and microRNA-30a-5p significantly increased in all PFOS exposed groups, except microRNA-16 in 50 µM PFOS.

## 3. Discussion

The present study has investigated the effect of epigenetic regulations on PFOS induced neurotoxicity by using in vitro tests with the human neuroblastoma cell line. The results showed that PFOS can injure SK-N-SH cells, decrease the expression of BDNF, result in different methylation status of BDNF promoter I and IV, change the DNMTs expression level, and increase the expression of microRNA-16, microRNA-22, and microRNA-30a-5p, which are related to the expression of BDNF. This evidence shows PFOS can induce the neurotoxicity through adjusting the methylation status of the BDNF promoter and increasing the expression of microRNAs related to the expression of BDNF.

PFOS is a ubiquitous persistent organic pollutant and that is widely distributed in the environment and in living organisms. The neurotoxicity of PFOS has received more concern compared with various toxic effects due to the ability to cross the blood brain barrier. Li reported that PFOS can induce SH-SY5Y cell apoptosis and injure the BDNF/TrkB/CREB signaling pathway [11]. In our study, the morphology analysis showed that PFOS exposure could lead to significant morphological abnormalities and reduce cell viability, and result in neurotoxicity in SK-N-SH cells. Chen reported that PFOS exposure could reduce learning ability in nematodes [2]. Johansson observed that prenatal exposure to PFOS led to disturbed behavior in the adult animal [24]. Our previous studies also indicated that prenatal exposure to PFOS could lead to an impairment of cognitive function connected with long-lasting changes in the expression of Synaptophysin and Synapsin1 and Synapsin2, as well as damage to the synaptic ultrastructure in rat hippocampi [25,26]. BDNF plays an important role in the development of neuro and synaptic plasticity by modulating the synthesis of synaptic proteins and enhancing the effect of synaptic necessary precursors for maintain neuronal function [9]. Oh reported that the decrease of BDNF would lead to cerebral atrophy, underlie age-related synaptic loss, cognitive function decline, and in particular, increase risk for psychiatric disease [27]. Our result also indicated that PFOS exposure reduced the expression of BDNF not only in mRNA level, as well as the protein level of BDNF. In present, a lot of studies have suggested that the BDNF binds to TrkB (Tyrosine kinase B) and activate downstream cell signaling in order to promote growth and survival, and maintain the plasticity of neural cells [28,29]. BDNF plays an important role in maintaining the structure and function of the nervous system. This evidence show that the BDNF decrease may be a part of mechanism of neurotoxicity induced by PFOS exposure.

However, it is still unclear about the mechanism of BDNF decrease induced by PFOS. The expression of BDNF is controlled by many factors such as epigenetic regulation. Epigenetic regulation plays a critical role in changing the expression of BDNF induced by environmental factors and in neurodegenerative disorders [30]. Recently, miRNAs have been regarded as a critical genomic regulator which plays an important role in the development of the nervous system and contributing to the correct calibration of neuronal gene expression. Silio prediction and reporter system indicated that the level of BDNF, a central node in the miR-mRNA regulatory network, can be post-transcriptionally modulated by upregulated microRNA-30a-5p [31]. Yun reported that cisplatin can downregulate the expression of BDNF through increasing microRNA-16 to inhibit the proliferation of SH-SY5Y cells in vivo and vitro [32]. Our previous study indicated that PFOS most likely acted through microRNA-22 inhibiting the expression of BDNF in SH-SY5Y cells [11]. The function of microRNA-16, microRNA-22, and microRNA-30a-5p, especially their influence on BDNF expression in neurotoxicity induced by PFOS need further academic research. Many emerging data have proved that increasing or decreasing the expression of special miRNAs can be result in different effects in neural cells [33]. Consistent with preceding results, our results suggested that incubation with PFOS significantly increase the levels of microRNA-16, microRNA-22, and microRNA-30a-5p in SK-N-SH cells after 48 h treatment. These results have identified that PFOS may significantly reduce the expression of BDNF through increasing the level of microRNA-16, microRNA-22, microRNA-30a-5p, and lead to neurotoxicity in SK-N-SH cells.

DNA methylations are considered to be another epigenetic modification, which are closely related to the pathogenesis of psychiatry. A large number of studies have proved the relationship between *BDNF* gene methylation level and certain psychological diagnoses. DNA methylation mechanisms have been shown to be important for BDNF expression regulation. Adverse environmental exposures could change the BDNF promoter methylation and inhibited the expression [34]. Early maltreatment could produce persisting methylation changes of BDNF promoter IV and decreased *BDNF* gene expression in prefrontal cortex of the adult [35]. The present study showed that PFOS exposure could lead to the methylation status change of BDNF promoter I and IV, which may disturb the expression of BDNF. Interestingly, PFOS exposure led to the hypermethylation of twentieth 5′CpG3′ site in promoter IV in our studied DNA region. However, whether this CpG site is a vital site for BDNF activity and expression regulation is not clear and needs testifying in future study. In addition, because bisulfite sequencing can only detect a short DNA fragment methylation level, whether PFOS may cause DNA methylation change in other part of the BDNF promoter needs to be affirmed. DNMTs play a critical role in learning and memory formation through regulation of BDNF expression and activity. Hypermethylation of hippocampal BDNF is involved in neonatal sevoflurane exposure-induced cognitive impairments via DNMTs mediated [36]. Maternal separation increased DNA methyltransferases expression in the nucleus accumbens of infant and adult rats, and promotes the development of cocaine-induced behavioral sensitization in adulthood [37]. Sui reports showed that epigenetic regulation of *BDNF* might underlie the mechanisms of synaptic plasticity and memory retention in the medial prefrontal cortex [38]. DNA methylation maintenance enzyme Dnmt1 has a high affinity for hemi-methylated DNA in vitro and plays an important role in the replicating phase to maintain DNA methylation following DNA synthesis [39]. However, de novo DNA methyltransferase DNMT3a and DNMT3b establish novel patterns of DNA methylation, and prefer to bind unmethylated DNA in vitro [40]. In our study, the mRNA and protein levels of DNMT1 significantly decreased after PFOS exposure for 48 h. On the contrary, PFOS exposure increased the mRNA and protein level of DNMT3b, but did not change the expression of DNMT3a, except for protein level in 50 μM PFOS group. These DNMTs expression changes may be related to the change of methylation status in BDNF promoter I and IV. These may play an important role in BDNF decrease induced by PFOS exposure.

## 4. Materials and Methods

### 4.1. Reagents and Chemicals

Perfluorooctane sulfonate (PFOS, purchased from Sigma-Aldrich, Shanghai, China) was dissolved in dimethyl sulfoxide (DMSO, purchased from Sigma-Aldrich, Shanghai, China) and stored at −20 °C. The PFOS solution was then diluted to the desired concentrations. Other chemicals used in the present study were commercially available and of appropriate grades.

### 4.2. Cell Culture and PFOS Treatment

SK-N-SH cells (purchased from Cell Bank of Life Sciences, Shanghai, China) were cultured with MEM (HyClone, Logan, UT, USA) supplemented with 10% FBS (ScienCell, Shanghai, China), 100 U/mL penicillin, and 100 μg/mL streptomycin in 5% CO_2_ at 37 °C. Cells were passaged every 2 to 3 days as follows: the adherent cells were washed with PBS, added into 1 mL 0.25% trypsin solution (Biosharp, Hefei, China), and then fresh medium was added, and cells were isolated by blowing tubes, finally dispensed into new flasks. Cells were seeded on 6-well-plates, 96-well-plates, or flat bottoms, and maintained in an incubator with 5% CO_2_ at 37 °C for 24 h until they adhered completely, and then incubated with different concentrations of PFOS or control medium (0.1% DMSO) for future experiments.

### 4.3. Morphological Study and Cell Viability Assay (MTT)

After the SK-N-SH cells had grown in the 6-well-plates for 24 h, they were treated with PFOS (50, 100, 150, 200, and 250 µM) or the control medium (0.1% DMSO) for 48 h. Then the morphology of SK-N-SH cells was examined and recorded with an inverted microscope (Olympus CKX41, Tokyo, Japan) after a 48-h exposure to the control medium (0.1% DMSO) or PFOS. Viability of cells was determined by MTT assay. After the SK-N-SH cells had grown in the 96-well-plates at 9 × 10^3^ cells/μL for 24 h, they were treated with PFOS for 48 h. And then, MTT reagents were added to the 96-well-plates and cells continued to incubate at 37 °C for 4 h. Discarding the supernatant and adding 150 μL DMSO per well, the UV-visible absorbance was measured at 450 nm using an enzyme-linked immunosorbent assay plate reader (BIO-TEK ELX-800, Winooski, VT, USA) within 10 min.

### 4.4. RNA Extraction and cDNA Synthesis

According to the manufacturer’s instructions, total RNA was extracted using TRIZOL (Invitrogen, Guangzhou, China). The nucleic acid purity was evaluated by the ratio of the optical densities of the RNA samples at 260 nm and 280 nm, and based on the absorbance at 260 nm the total RNA concentrations were evaluated. According to the manufacturer’s instructions, cDNA synthesis for miRNAs and mRNA was performed with 2 μg of total RNA by using the miScript II RT Kit (Qiagen, Guangzhou, China) and Thermo Scientific RevertAid First Strand cDNA Synthesis Kit (Thermo, Shanghai, China). cDNA was stored at −80 °C.

### 4.5. Real-Time Quantitative PCR (Q-PCR)

The expression of BDNF, DNMT1, DNMT3a, and DNMT3b at mRNA levels were analyzed with SYBR Green Supermix (Bio-Rad, Irvine, CA, USA) by following the manufacturer’s instructions. The sizes of products and their corresponding primer sequences used in this study were listed in Table 1. The gene specific primers were designed and synthesized by Guangzhou Invitrogen Corporation. The QPCR reaction was performed in the iQ5 Multicolor Real-Time PCR Detection System (Bio-Rad, Irvine, CA, USA) in duplicates with 25 ng cDNA and Sybgreen Universal PCR Master Mix in a total volume of 25 μL. The QPCR procedure included an initial denaturation at 95 °C for 10 min, followed by 40 cycles of 95 °C for 15 s (denaturation), 55 °C for 30 s (annealing), and 72 °C for 30 s (extension). The expression levels of microRNA-16, microRNA-22, and microRNA-30a-5p were analyzed with the miScript SYBR Green PCR Kit and miScript Primer Assays (Qiagen, Guangzhou, China) according to the manufacturer’s instructions. Each sample was run in triplicate for the target and reference genes, along with a single NRT (no reverse transcriptase) negative control with a total reaction volume of 25 μL. β-Actin and U6 were used as endogenous reference genes, which were uniformly expressed among all samples (cycle threshold standard deviation less than 0.5) for mRNA and microRNA, respectively. A dissociation curve analysis was performed for each gene to examine the amplification of the non-targeted fragment. Only one peak was observed for each reaction, indicating that only the target gene was amplified. The 2^−ΔΔ*C*t^ (*C*_t_, cycle threshold) method was used to calculate the fold changes in relative target gene expression [41].

### 4.6. Enzyme Linked Immunosorbent Assay (ELISA)

Enzyme Linked Immunosorbent Assay (ELISA) was used to analyze the protein levels of BDNF. The SK-N-SH cells cultured in 6-well plates were treated with PFOS (0, 50, 100, and 150 µM) for 48 h, and then the supernatants were collected through centrifuged at 1000 rpm for 3 min at 4°C. The supernatants were added to the Human BDNF ELISA Kit (ExCell Biology, Inc., Shanghai, China) which were coated with a primary, the antibody for human BDNF and incubated at 37 °C for 2 h, then the ELISA plate was washed with scrubbing solution five times. The secondary biotinylated anti-human BDNF antibody was added, followed by the addition of HRP-conjugated streptavidin. And then, TMB substrate solution was added to the wells as the developing agent, and the color development was in proportion to the amount of bound BDNF. Finally, the stop solution was added to change the color from blue to yellow, and the optical density was detected at 450 nm (BIO-TEK ELX-800, Winooski, VT, USA). All standards or samples were run in triplicate and the standard curve was run for each assay.

### 4.7. Western Blot Assays

The SK-N-SH cells had grown in flat bottoms at 2.5 × 10^3^ cells/cm^2^ for 24 h, and then incubated with PFOS (50, 100, 150 μM) or control medium (0.1% DMSO) for 48 h. Cells were washed with PBS (4°C) three times and lysed in RIPA buffer. Homogenates were clarified by centrifugation at 12,000× *g* for 15 min at 4°C. The concentrations of total cellular proteins were detected by bicinchoninic acid (BCA) protein assay kit (Beyotime, Shanghai, China). Samples with equal amounts of protein were solubilized in loading buffer (0.5 mM Tris, pH 6.8, 1% SDS, 5% DTT, 0.01% bromophenol blue, and 4% lycerinum) and separated by electrophoresis at 80 V in stacking gel and 120 V in separation gel. The power was turned off until the bromophenol blue reached the bottom of the electrophoresis chamber. Then, gels were transferred to polyvinylidene difluoride (PVDF) membranes and incubated with the primary antibodies for a night: anti-DNMT1, anti-DNMT3a, anti-DNMT3b (Abcam, Cambridge, UK), anti-actin (Sigma-aldrich, Shanghai, China). And then, HRP (Proteintech, Chicago, IL, USA) were used as secondary antibodies. Finally, the PVDF with SuperECL Plus (Beyotime, Shanghai, China) was incubated for 3 min and developed in Gel Imaging System (Tanlon, shanghai, China).

### 4.8. Determination of the Status of BDNF Promoter Methylation

The bisulfite reaction was carried out according to the protocol provided by Cells-to-CpG™ Bisulfite Conversion Kit (Thermo, Shanghai, China). The converted DNA was amplified by the polymerase chain reaction (PCR) using GoTaq^®^ Hot Start Green Master Mix (Promega, Madison, WI, USA) with BDNF promoter I (product size, 446 bps) forward primer: 5′-AGTTTTTGTAGGATGAGGAAGTGGT-3′ and reverse primer: 5′-ACCATCATAACTAAAAATCTCCAACC-3′. For BDNF promoter IV (product size, 406 bps), the forward primer was 5′-AGTTTGTTGGGGTTGGAAGTG-3′, the reverse primer was 5′-ATACCC(A/G)ATATATACTCCTTCTATTCTACA-3′. PCR amplifications were performed in 30 μL reaction mixtures containing pooled 2 μL of bisulfite-treated genomic DNA, under the following reaction conditions: 5 min at 95 °C, 10 cycles for 30 s at 94 °C, 30 s at 60 °C down 50 °C and 30 s at 72 °C, 25 cycles for 30 s at 94 °C, 30 s at 50 °C and 30 s at 72 °C and finally at 60 °C for 30 min. The PCR products were separated on 2% agarose gels and purified by gel extraction using the Wizard^®^ SV Gel and PCR Clean-Up System (Promega, Madison, WI, USA), then cloned into a pCRII vector using TA Cloning Kit Dual Promoter (Invitrogen, Grand Island, NY, USA). Plasmids DNA from 10 colonies were prepared using TIANprep Mini Plasmid Kit (TIANGEN, Beijing, China) and sequenced.

### 4.9. Statistical Analysis

All data are presented as the Mean ± SD, and statistical analysis were performed with one-way ANOVA followed by LSD and Dunnett’s T3 test using SPSS 17.0 (SPSS Inc., Chicago, IL, USA). A *p* value < 0.05 was considered statistically significant in all experiments.

## 5. Conclusions

The present study has revealed that PFOS exposure can induce the decrease of cell viability in SK-N-SH cells and the expression of BDNF. There is also a correlation between the decrease of BDNF level and the increased mRNA levels of microRNA-16, microRNA-22, and microRNA-30a-5p. Moreover, exposure to PFOS can reduce DNMT1 expression, but increases the protein levels of DNMT3b. This may contribute to the methylation status change of BDNF promoter I and IV. The present study has identified that the epigenetic regulation of the *BDNF* gene may be involved in PFOS-induced neurotoxicity. 

## Figures and Tables

**Table 1:** Primer sequences for quantitative real-time PCR.

Gene Name	Primer	Product (bp)
*BDNF*	Forward: AGCTGAGCGTGTGTGACAGTATTAG	129
	Reverse: ATTGCTTCAGTTGGCCTTTTGATAC	
*DNMT1*	Forward: CGACTACATCAAAGGCAGCA	161
	Reverse: TGGACTTGTGGGTGTTCTCA	
*DNMT3a*	Forward: GCATTGTGTCTTGGTGGATG	191
	Reverse: GACCTCGTAGATGGCTTTGC	
*DNMT3b*	Forward: AGGATGGGAAGGAGTTTGGA	114
	Reverse: ACATAGCCTGTCGCTTGGAG	
*Actin*	Forward: TGAAGGTGACAGCAGTCGGTT	126
	Reverse: ACTTCCTGTAACAACGCATCTCATA	
